# Fractionated breath condensate sampling: H_2_O_2 _concentrations of the alveolar fraction may be related to asthma control in children

**DOI:** 10.1186/1465-9921-13-14

**Published:** 2012-02-14

**Authors:** Jordis Trischler, Nick Merkel, Stephanie Könitzer, Christina-Maria Müller, Susanne Unverzagt, Christiane Lex

**Affiliations:** 1 Department of Paediatrics, University Children's Hospital Halle (Saale), Germany; 2 Martin-Luther-University Halle-Wittenberg, Halle (Saale), Germany; 3 Institute for Medical Epidemiology, Biostatistics and Informatics, University Halle (Saale), Germany

**Keywords:** Paediatric asthma, exhaled airway markers, oxidative stress

## Abstract

**Background:**

Asthma is a chronic inflammatory disease of the airways but recent studies have shown that alveoli are also subject to pathophysiological changes. This study was undertaken to compare hydrogen peroxide (H_2_O_2_) concentrations in different parts of the lung using a new technique of fractioned breath condensate sampling.

**Methods:**

In 52 children (9-17 years, 32 asthmatic patients, 20 controls) measurements of exhaled nitric oxide (FE_NO_), lung function, H_2_O_2 _in exhaled breath condensate (EBC) and the asthma control test (ACT) were performed. Exhaled breath condensate was collected in two different fractions, representing mainly either the airways or the alveoli. H_2_O_2 _was analysed in the airway and alveolar fractions and compared to clinical parameters.

**Results:**

The exhaled H_2_O_2 _concentration was significantly higher in the airway fraction than in the alveolar fraction comparing each single pair (p = 0.003, 0.032 and 0.040 for the whole study group, the asthmatic group and the control group, respectively). Asthma control, measured by the asthma control test (ACT), correlated significantly with the H_2_O_2 _concentrations in the alveolar fraction (r = 0.606, p = 0.004) but not with those in the airway fraction in the group of children above 12 years. FE_NO _values and lung function parameters did not correlate to the H_2_O_2 _concentrations of each fraction.

**Conclusion:**

The new technique of fractionated H_2_O_2 _measurement may differentiate H_2_O_2 _concentrations in different parts of the lung in asthmatic and control children. H_2_O_2 _concentrations of the alveolar fraction may be related to the asthma control test in children.

## Background

Asthma is a chronic inflammatory disease that is predominantly characterised by inflammatory processes of the airways. However, studies have shown that alveoli are also subject to pathophysiological changes and might play a central role for asthma control and severity of the disease [1-4]. In childhood, inflammation is mostly caused by allergic and eosinophilic changes [5], and so far, alveolar involvement has been demonstrated mainly by measurement of alveolar nitric oxide, a marker of eosinophilic inflammation. However, reactive oxygen species (ROS) like hydrogen peroxide (H_2_O_2_) seem to play a role in the pathophysiology of childhood asthma [6-8]. Jöbsis et al. demonstrated that in children with asthma, overall exhaled H_2_O_2 _is elevated compared to controls, but to date there is no account of the contribution of the alveoli to these elevated H_2_O_2 _concentrations [9].

Exhaled breath condensate (EBC) is a well-known method to collect inflammation mediators and other soluble particles in exhaled breath [10]. Until recently it has only been possible to detect markers like H_2_O_2 _in unfractionated breath condensate, which did not allow detecting the origin of production. A new method of fractionated sampling now provides the ability to collect condensate from different parts of the lung. By measuring ROS like H_2_O_2_, it may be possible to locate the origin of active inflammation and therefore weigh the contribution of the alveoli to the severity of asthma and asthma control. Whereas results for fractionated H_2_O_2 _measurements in adults with COPD are published, use of this technique in asthma and in children has not yet been described [11].

One of the aims of this study was to apply the new technique of fractionated breath condensate sampling in children. Through the new technique, the principal aim was to compare H_2_O_2 _in different condensate fractions in asthma. Our primary hypothesis was that airway H_2_O_2 _concentrations are significantly higher than alveolar concentrations. In addition we aimed to correlate these results with data of exhaled nitric oxide (FE_NO_), lung function measurements and the asthma control test (ACT).

## Methods

### Subjects

Asthmatic patients (aged 9-17 years) were recruited from the asthma clinic of the Children's University Hospital Halle. Asthma was diagnosed clinically when children had episodic cough, breathlessness and wheeze responsive to bronchodilators according to International and American Thoracic Society (ATS) criteria.

Healthy non-atopic controls with no history of chronic cough, wheezing or other pulmonary symptoms and without any chronic disease involving the immune system (e.g. Crohn's disease, Diabetes and rheumatic diseases) were recruited in various outpatient clinics.

Subjects who were active smoking or had an acute respiratory infection during the previous two weeks were excluded.

### Study design

First, subjects underwent clinical examination, an asthma questionnaire was filled out and atopic sensibilisation was tested. Atopic sensibilisation was diagnosed by RAST or prick test, atopy was defined by a serum-specific IgE > 0.34 kU/L or a positive skin prick test (wheal > 2 mm larger than negative control) to at least one antigen (D. pteronyssinus, D. farinae, cat, dog, grass pollen, birch pollen, Aspergillus fumigatus). Afterwards subjects completed FE_NO _measurement, lung function testing, and collection of fractionated EBC in this chronological order. Subjects completed the study protocol within 4 hours.

This cross-sectional study was approved by the local ethics committee of the Martin-Luther-University Halle-Wittenberg. Written consent was obtained from the participant's parents and age-appropriate consent from the children themselves.

Power analysis: We assumed a probability of 70% achieving higher values of airway concentrations compared to alveolar concentrations as clinical relevant. With this probability and a significant level of α = 0.05 a sample size of 44 subjects is sufficient to achieve a power of 90% in a two-sided Wilcoxon signed-rank test.

### Asthma questionnaire

To evaluate disease control for children 12 years or older the Asthma Control Test (ACT) was used, for children younger than 12 years the Childhood Asthma Control Test was used [12,13]. Due to different scoring systems, these tests were not comparable. Parents and subjects were asked for passive smoking histories.

### Lung function tests

Bodyplethysmography and spirometry (Masterlab, Jaeger, Würzburg, Germany) were performed for measurement of FEV_1 _(forced expiratory volume in 1 second), VC (vital capacity), MEF_25 _(maximal expiratory flow at 25% of VC), MEF_50 _(maximal expiratory flow at 50% of VC), MEF_75 _(maximal expiratory flow at 75% of VC), ITV (intrathoracic volume), TLC (total lung capacity), RV%TLC (residual volume- total lung capacity ratio) and sR_aw, tot _(specific airway resistance) as previously reported for all subjects [14].

### FE_NO _-Measurement

FE_NO _was measured for each subject using NiOX MINO^® ^(Aerocrine, Sweden), at a flow rate of 50 mL/s, according to the manufacturer's instructions.

### Collection of fractionated exhaled breath condensate

Exhaled breath condensate was collected according to the current ATS/ERS guidelines using ECoScreen 2^® ^(Filt GmbH, Germany). This new system, constructed to collect fractionated breath condensate, passes captured air through two different chambers depending on the settings for the single exhaled breath volume and the threshold between the airway and alveolar fraction. The collector includes a spirometer for measuring exhaled volumes and peak flows.

For this study, the separation threshold has been set at one third of the exhaled breath volume based on the results of Möller et al. [11] which showed, based on exhaled CO_2 _profiles, the starting point of the alveolar plateau is at approximately one third of the exhaled breath volume. Exhaled breath volume was set to 1200 mL for subjects ≥ 60 kg, leading to sampling volumes of 400 mL for fraction 1 and 800 mL for fraction 2. Fraction 1 is thought to represent the airway fraction and fraction 2 the alveolar fraction. In children less than 60 kg the exhaled breath volume was proportionately weight-adjusted, with 60 kg and 1200 mL set as 100%. Subjects wore a nose clip to allow only orally exhaled breath condensate to be collected. Patients were asked to breathe slowly and regularly with an increased tidal breathing. Standardised breathing patterns were enforced by asking patients to breathe out until they heard a beep from the collecting machine.

The sampling period was ended after the total gas sampling volume was 300 L or 200 L depending on the subjects' tolerance. Sampling times and mean peak flow values were recorded. Breath condensate volumes for each fraction were measured and condensate immediately transferred for further analysis.

### Analysis of H_2_O_2 _concentrations

For further analysis of H_2_O_2 _concentration, ECoCheck (Filt GmbH, Germany) was used as described by Gerritsen et al. [15]. With this device, the H_2_O_2 _value is obtained by a specific reaction of the substance with oxidase that is followed by amperometric detection. Values can be measured in a range of 15 to 10 000 nmol/L according to the manufacturer's instructions.

### Data analysis

Data was processed using SPSS (version 12.0). Values are expressed as median and interquartile range (IQR). Correlations were measured using the Spearman rank correlation. Comparisons between groups were made using the Mann-Whitney-U test. Differences of H_2_O_2 _concentration between the fractions were analysed by the Wilcoxon signed-rank test, comparing each single related sample pair (paired difference test). Results with p < 0.05 were considered statistically significant.

## Results

### Patient characteristics

32 asthmatic and 20 non-asthmatic children were included in this study. Patient characteristics are shown in table 1. Approximately half of the asthmatic patients were prescribed inhaled corticosteroids (ICS) with a median dosage of 200 μg (IQR 0-400 μg).

**Table 1:** Patients characteristics

	Asthmatic patients	Controls	p
**n**	32	20	

**Age (yr)**	12.5 (11.0-15.0)	13.5 (12.0-15.8)	ns

**Female (n)**	11	12	ns

**Atopy (n)**	30	0	

**Passive smoking**	9	7	ns

**Treatment**			
**ICS (n)**	17		
Beclomethasone			
equivalent dosage (μg)	200 (0-400)		
Budesonide (n)	13		
Fluticasone (n)	4		
**Bronchodilator (n)**	12		
Salbutamol	4		
Formeterol	8		

**Montelukast (n)**	3		

**FEV_1 _(% pred.)**	105 (97-118)	107 (101-116)	ns

**VC (% pred.)**	103 (96-112)	101 (89-99)	ns

**MEF 25 (% pred.)**	104 (83-115)	100 (79-113)	ns

**MEF 50 (% pred.)**	94 (76-126)	104 (84-124)	ns

**MEF 75 (% pred.)**	92 (75-117)	125 (91-158)	ns

**TLC (% pred.)**	98 (85-104)	92 (87-100)	ns

**ITV (% pred.)**	101 (82-113)	101 (86-121)	ns

**RV/TLC (% pred.)**	88 (71-98)	92 (73-99)	ns

sR_aw, tot _(kPa*s)	0.67 (0.50-0.91)	0.50 (0.44-0.63)	ns

FE_NO _(ppb)*	17.0 (8.0-39.3)	9.0 (7.0-11.0)	0.001

**ACT (points)**			
**< 12 years (n = 11)**	25 (22-26)		
**≥ 12 years (n = 21)**	23 (21-25)		

**H_2_O_2 _concentrations airway fraction (nmol/L)**	290 (155-505)	310 (115-555)	ns

**H_2_O_2 _concentrations alveolar fraction (nmol/L)**	220 (140-460)	180 (120-320)	ns

Values as medians (IQR) or numbers, as appropriate; ns: not significant

Asthmatic patients had significantly higher FE_NO _values than controls (median 17.0 ppm (8.0-39.3) vs. 9.0 ppm (7.0-11.0), p = 0.001). There were also significant lower FE_NO _values in asthmatic patients using ICS than in asthmatic patients without regular usage of ICS (median (IQR) 12.0 ppb (6.5-21.5 ppb) vs. 28.0 ppb (17.0-44.0 ppb), p = 0.015).

### Exhaled H_2_O_2 _concentrations and sampling parameters

14 of 32 asthmatic patients and 10 of 20 control subjects succeeded in collecting breath condensate with 300 litres of total gas volume, 18 of 32 patients and 10 of 20 controls only succeeded in collecting 200 litres. Details of the sampling times, peak flow values and sampled condensate volumes are presented in table 2. There was no significant difference in the alveolar and airway H_2_O_2 _concentrations whether the total gas sampling volume was 200 l or 300 l. Therefore the following statistical calculations were done for asthmatic patients and controls irrespective of the total gas sampling volumes achieved.

**Table 2:** Results of the fractionated exhaled breath condensate sampling parameters

	*Asthmatic patients*			*Controls*		
**Total gas sampling volume (L)**	**200**	**300**	**p**	**200**	**300**	**p**

**n**	18	14		10	10	

**Sampling time (min)**	8.6 (6.9-11.7)	12.8 (9.3-16.1)	0.01	9.2 (7.9-10.6)	9.7 (8.1-11.1)	ns

**Peak flow (L/s)**	2.2 (1.5-2.7)	2.0 (1.4-2.2)	ns	2.1 (1.8-2.6)	2.0 (1.5-2.4)	ns

**Condensate volume** **airway fraction (μL)**	410 (293-625)	825 (450-1050)	0.02	400 (303-540)	600 (488-763)	0.007

**Condensate volume** **alveolar fraction (μL)**	1275 (988-1650)	2000 (1400-2400)	0.07	1200 (925-1838)	2150 (1670-2400)	0.009

**H_2_O_2 _concentration fraction 1 (nmol/L)**	290 (95-515)	310 (160-640)	ns	250 (95-545)	320 (155-615)	ns

**H_2_O_2 _concentration fraction 2 (nmol/L)**	270 (155-475)	190 (115-405)	ns	140 (120-290)	250 (135-450)	ns

Values as medians (IQR) or numbers, as appropriate; ns: not significant

H_2_O_2 _concentrations were below the detection limit (15 nmol/L) in three samples of fraction 1 (airway fraction) and in one sample of fraction 2 (alveolar fraction). In these 4 cases we used values of 0 nmol/L for statistical analysis, but only performed nonparametric tests. Furthermore H_2_O_2 _concentrations could not be obtained in 2 samples of the airway fraction because sample analysis curves were not acceptable.

No significant correlation was found between H_2_O_2 _concentrations of both fractions and the peak flow values during sampling breath condensate.

### Comparison of H_2_O_2 _concentrations between the airway and the alveolar fraction

**Figure 1:**
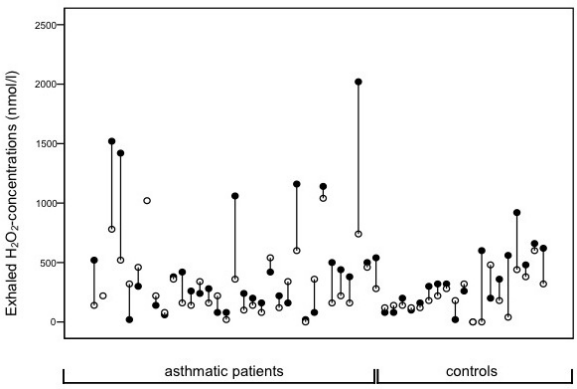
**Paired exhaled H_2_0_2 _concentrations (● = airway fraction, ○ = alveolar fraction) of asthmatic children and controls**. The exhaled H_2_O_2 _concentration was significantly higher in the airway fraction than in the alveolar fraction comparing each single pair (p = 0.003).

Comparing H_2_O_2 _concentrations between the two fractions for each related single pair we found that H_2_O_2 _concentrations were significantly higher in the airway fraction than in the alveolar fraction. P-values using the Wilcoxon signed-rank test were 0.003, 0.032 and 0.040 for the whole study group, the asthmatic group and the control group, respectively. Medians and IQRs are shown in tables 1 and 2, detailed values of each single patient are shown in Figure 1. P-values were 0.034 for the subgroup of patients with a sampling volume of 200 L and 0.053 for patients with a sampling time of 300 L.

There was no significant difference in H_2_O_2 _concentrations between asthmatic patients and controls. However, extremely high values above 1000 nmol/L were only present in asthmatic patients (n = 6) and not in controls (Figure 1).

### Correlation of exhaled H_2_O_2 _concentrations to passive smoking

There was no significant difference in H_2_O_2 _concentrations whether the patients or their parents reported second hand smoke exposure.

### Correlation of exhaled H_2_O_2 _concentrations to asthma questionnaire

**Figure 2:**
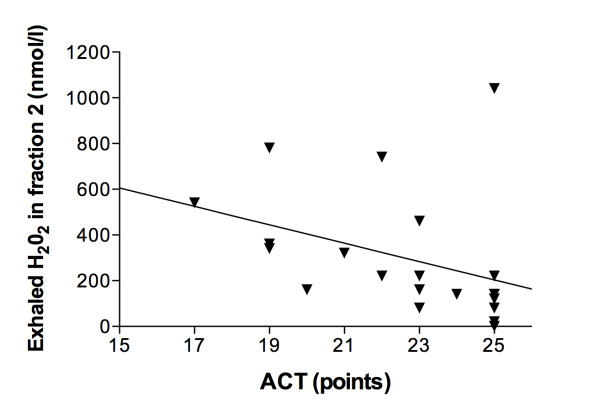
**Spearman's correlation between the asthma control test (ACT) in points and H_2_0_2 _concentrations in the alveolar fraction of asthmatic children 12 years and older**. The asthma control test correlated significantly with the H_2_O_2 _concentrations in the alveolar fraction (r = -0.606, p = 0.004).

H_2_O_2 _concentrations in the alveolar fraction, but not in the airway fraction, were negatively correlated to the asthma control test for children 12 years or older (r = -0.606, p = 0.004, Figure 2). One outlier showed very high H_2_O_2 _concentrations with a perfect ACT score. There was no correlation with the asthma control test for younger children.

Furthermore H_2_O_2 _concentration of both fractions did not differ based on prescribed ICS use.

### Correlation of exhaled H_2_O_2 _concentrations to lung function parameters

We found no correlation between H_2_O_2 _concentrations and lung function parameters as described above. In particular, there was no correlation between H_2_O_2 _concentrations in the alveolar fraction and lung function parameters, which suggest an involvement of the distal airways including MEF_75 _(% pred.) and RV/TLC (% pred.).

### Correlation of exhaled H_2_O_2 _concentrations to FE_NO _values

There was no positive correlation detected between H_2_O_2 _concentrations and FE_NO _values in asthmatic patients, regardless of which fraction was measured. Furthermore, in 5 out of 6 patients with very high airway H_2_O_2 _concentrations (above 1000 nmol/L), low NO values (< 20 ppb) were measured. Looking at alveolar H_2_O_2 _concentrations, the results were similar; both of the two patients who had H_2_O_2 _values above 1000 nmol/L had low NO values (< 20 ppb).

## Discussion

In this study we showed for the first time a significant difference between H_2_O_2 _concentrations in two different lung compartments of asthmatic children. The H_2_O_2 _concentration in the airway fraction was significantly higher than in the alveolar fraction. Additionally, we demonstrated a significant correlation of asthma control and H_2_O_2 _concentrations in the alveolar fraction.

Whereas the role of alveolar NO as a non-invasive marker of inflammation in asthma has been shown, this study contributes to the assessment of fractionated H_2_O_2 _in this setting. Whereas results of fractionated H_2_O_2 _detection in COPD are published [11], results in asthma and in children are still missing. The new technique of fractionated H_2_O_2 _measurement allows a differentiation of H_2_O_2 _concentrations in different parts of the lung. Our findings demonstrate a significantly lower concentration of H_2_O_2 _in the alveolar fraction than in the airway fraction of asthmatic patients. These results are in accordance to those of Möller et al. who found lower H_2_O_2 _concentrations in the alveolar fraction of adult COPD patients [11]. The distribution pattern of H_2_O_2_, showing lower values in the alveolar fraction than in the airway fraction, is consistent with the distribution of other inflammation markers like NO and IL-4 [3,16].

For children 12 years and older, we showed for the first time that worse asthma control correlates with higher H_2_O_2 _concentrations in the alveolar fraction. There were outliers, but the results still show a significant trend towards higher concentrations in suboptimally controlled asthma. The relationship between lung inflammation in general and an increased production of reactive oxygen species is well known [8,17]. However, our findings support the assumption that inflammation in the alveoli may play an important role in asthma control.

Growing evidence shows the importance of distribution patterns of inflammation rather than total values for a more complex understanding of asthma. Eosinophils as a marker for inflammation have been found in all parts of the airways, but the amount of eosinophils in peripheral airways seems to correlate with asthma severity [1]. Exhaled NO has been frequently linked to eosinophilic inflammation, and several studies show that high alveolar NO values correlate with worse asthma control [3,17].

There have been studies that tried to link elevated alveolar inflammation to peripheral lung function parameters. Van Veen et al. could demonstrate a correlation between alveolar NO and RV/TLC (% pred.), a marker for distal air trapping [18]. Also TLC and TGV have been positively correlated to distal lung inflammation measured by eosinophilic alveolar inflammation [19]. Correlations of distal inflammation to other lung function parameters like FEV_1 _and MEF_25-75 _are more conflicting [19]. In our study, we could not find a correlation between the mentioned lung function parameters and the alveolar fraction of H_2_O_2_. However, lung function parameters in children are mostly within normal values and do not correlate with asthma severity [20], especially since we excluded patients with acute infections.

We did not find any significant relation between H_2_O_2 _concentrations of the airway fraction and FE_NO_, mainly representing the NO deriving from the airway fraction [3]. A similar discordant behavior between FE_NO _and EBC 8-isoprostane, another marker of airway oxidative stress [21], has been observed in asthmatic children with exercise-induced bronchoconstriction [22]. This may be due to different types of airway inflammation, which are represented by the two values. As shown by us in a former study, FE_NO _correlates strongly with the amount of bronchoalveolar lavage (BAL) eosinophils and is generally thought of as a marker for eosinophilic inflammation [23]. Sources of H_2_O_2 _are thought to be more diverse compared to those of FE_NO_, since the producing superoxide dismutase can be found in a range of cells, i.e. macrophages, alveolar type II cells and the amount of H_2_O_2 _might be amplified by neutrophilic and eosinophilic peroxidases [24-26]. Since we found a significant correlation between alveolar H_2_O_2 _and asthma control, this implies the importance of measuring an additional biomarker, not only representing eosinophilic inflammation but oxidative stress. This assumption is strengthened by our findings that patients with very high H_2_O_2 _concentrations (above 1000 nmol/L) have mostly low FE_NO _values (< 20 ppb). Different phenotypes of inflammation may be measurable in paediatric asthma, providing additional information for assessing asthma control. Studies aiming at assessing other non-eosinophilic exhaled markers of airway inflammation including LTB4 [27] and volatile organic compounds [28] are required to reinforce the present data.

In our study there was a significant difference in FE_NO _values but not in H_2_O_2 _concentrations between steroid naïve asthmatic patients and asthmatic patients taking ICS, leading to the assumption that FE_NO _values, but not H_2_O_2 _concentrations may be suppressed by corticosteroids. This may be in accordance to the fact that Horvath et al. mostly found elevated H_2_O_2 _concentrations but normal FE_NO _values in their steroid-treated, unstable asthmatic group [29]. Unfortunately, to our knowledge no follow-up studies exist looking at H_2_O_2 _concentrations before and after corticosteroid prescription to support our findings. Also leukotriene receptor antagonists, which are widely used for asthma treatment [30], reduce FE_NO _concentrations in asthmatic children [31], but their effect on EBC hydrogen peroxide in asthmatic children is unknown and should be clarified. Likewise, future studies should establish whether measurement of hydrogen peroxide in the alveolar fraction of EBC might be useful for choosing the best pharmacological strategy in children with mild asthma [32].

We did not find any correlation between asthma control and H_2_O_2 _concentration in the group of children < 12 years. This may be due to the much smaller group of children (n = 11 vs. n = 21). Furthermore, assessment of asthma control for children < 12 years might be more difficult compared to children ≥ 12 years, because the integration of the parents' perception might cause a bias.

In this study we were the first to apply the new technique of fractionated breath condensate sampling to measure H_2_O_2 _concentrations in asthmatics and in children. We admit that the sampling technique in this study differed in between our study group, which might be a weakness of our study. Finally about half of the children succeeded in collecting 300 litres of gas volume, whereas the other half only reached 200 litres. However, there were no significant differences between H_2_O_2 _concentrations in both collecting groups (table 2).

A very difficult issue in collecting fractionated breath condensate sampling is the determination of the threshold between the alveolar and the airway fraction. In our study, we applied the threshold according to the mean gas sampling volumes of both fractions measured in the study of Möller et al. [11]. We admit that the one third/two third ratio we chose represents the volume relations in adult airways and may not be applied to the growing lungs of children. However, imaging studies show that the airway surface length/area ratio was linearly associated to alveolar surface/volume ratio in CT scans of 50 children from 0-17.2 years of age [33]. In the imaging based study by de Jong and colleagues, there is no over proportional growth of the alveolar volume, suggesting that the growth of the airways and the alveoli is closely linked.

Another problematic issue in separating airway and alveolar compartments is the possible inhomogeneous narrowing of the asthmatic airways. This could potentially lower alveolar volume values in children with less controlled asthma. We admit that the accuracy of the single test might be limited in our young subjects, since the threshold was not determined individually for each patient. Whether a capnograph based method instead of a volume based method for differentiation between both compartments may be applied in children and will reveal different results needs further investigations.

We were unable to include a flow restrictor into the experimental design of the machine to keep the flow constant during the exhalation. Schleiss et al. found out that H_2_O_2 _concentrations negatively correlated to the expiratory flow [34]. Concurrent with this, a recent publication by Gajdocsi et al. showed that H_2_O_2 _concentrations are lower during increased tidal breathing compared to tidal breathing [35]. In our study, patients had an increased tidal volume and therefore an increased flow during the first part of the exhalation. The measured H_2_O_2 _concentration could be falsely decreased since the expiratory flow was higher during collecting the airway fraction. We measured the expiratory peak flow in our study, but could not find any significant correlation of this value to the H_2_O_2 _concentrations of either fraction.

## Conclusion

In summary, this study showed for the first time that H_2_O_2 _concentrations in exhaled breath condensate were significantly higher in the airway fraction than in the alveolar fraction in asthmatic children and young adolescents. Only the H_2_O_2 _concentrations of the alveolar fraction correlated with asthma control in children 12 years and older suggesting that alveolar H_2_O_2 _plays a role in asthma control. However, whether fractionated exhaled H_2_O_2 _may be used as a non-invasive marker of alveolar involvement in asthmatics needs to be further investigated.

## Competing interests

The authors declare that they have no competing interests.

## Authors' contributions

CL conceived and coordinated the study and revised the manuscript. JT and NM carried out interpretation of the data, drafted the manuscript and participated in the study design. SK and CMM carried out the acquisition and analysis of the data. SU contributed as statistical advisor. All authors have read and approved the final manuscript.
